# Resting Heart Rate Does Not Predict Cardiovascular and Renal Outcomes in Type 2 Diabetic Patients

**DOI:** 10.1155/2016/6726492

**Published:** 2015-12-28

**Authors:** Vendula Bartáková, Linda Klimešová, Katarína Kianičková, Veronika Dvořáková, Denisa Malúšková, Jitka Řehořová, Jan Svojanovský, Jindřich Olšovský, Jana Bělobrádková, Kateřina Kaňková

**Affiliations:** ^1^Department of Pathophysiology, Medical Faculty, Masaryk University Brno, Kamenice 5, 62500 Brno, Czech Republic; ^2^Institute of Biostatistics and Analyses, Masaryk University Brno, Kamenice 126/3, 62500 Brno, Czech Republic; ^3^Department of Internal Medicine-Gastroenterology, University Hospital Brno, Jihlavská 20, 62500 Brno, Czech Republic; ^4^2nd Department of Internal Medicine, St. Anne's University Hospital, Pekařská 53, 65691 Brno, Czech Republic

## Abstract

Elevated resting heart rate (RHR) has been associated with increased risk of mortality and cardiovascular events. Limited data are available so far in type 2 diabetic (T2DM) subjects with no study focusing on progressive renal decline specifically. Aims of our study were to verify RHR as a simple and reliable predictor of adverse disease outcomes in T2DM patients. A total of 421 T2DM patients with variable baseline stage of diabetic kidney disease (DKD) were prospectively followed. A history of the cardiovascular disease was present in 81 (19.2%) patients at baseline, and DKD (glomerular filtration rate < 60 mL/min or proteinuria) was present in 328 (77.9%) at baseline. Progressive renal decline was defined as a continuous rate of glomerular filtration rate loss ≥ 3.3% per year. Resting heart rate was not significantly higher in subjects with cardiovascular disease or DKD at baseline compared to those without. Using time-to-event analyses, significant differences in the cumulative incidence of the studied outcomes, that is, progression of DKD (and specifically progressive renal decline), major advanced cardiovascular event, and all-cause mortality, between RHR </≥65 (arbitrary cut-off) and 75 (median) bpm were not found. We did not ascertain predictive value of the RHR for the renal or cardiovascular outcomes in T2DM subjects in Czech Republic.

## 1. Introduction

Elevated resting heart rate (RHR) has been associated with increased risk of all-cause mortality and cardiovascular (CV) events in healthy subjects as well as those with preexisting CV disease (CVD) including hypertension, acute myocardial infarction, and heart failure or left ventricular dysfunction by numerous epidemiologic studies and recently reviewed ones by Palatini and Julius [1] and Fox et al. [2]. In a recent study of Woodward et al., individual data from 112,680 subjects in 12 cohort studies were collected and an association between RHR above 65 beats/min (bpm) and the risk of both CV and all-cause mortality has been found independent of preexisting CVD [3]. Plausible pathophysiological mechanisms were reviewed by Lang et al. [4] and include, briefly, both indirect mechanisms related to autonomic dysregulation and those directly related to an increased heart rate per se (such as increased ischaemic burden and local haemodynamic forces adversely impacting on the endothelium and arterial wall).

Several studies focused on RHR in type 2 diabetic (T2DM) subjects. Stettler et al. found an association between RHR and all-cause mortality and CVD in a cohort of 302 T2DM patients [5]. Linnemann and Janka have identified an elevated RHR as a high risk for CV death in a cohort of 475 T2DM patients [6]. Hillis et al. found a relationship between baseline higher RHR and all-cause mortality, CV death, and major CV events (nonfatal myocardial infarction or nonfatal stroke) in a cohort of 11,140 T2DM patients; the increased risk associated with a higher baseline RHR was most obvious in patients with previous macrovascular complications [7]. Hillis et al. also extended the study on the same cohort of T2DM patients on the effect of RHR and microvascular complications (nephropathy and retinopathy) and reported an increased incidence and a greater progression of [8].

There are, however, fewer data on the relationship of RHR and renal events in diabetic subjects. Miot et al. studied a cohort of 1088 T2DM patients for the association of RHR with the incidence of composite CV and renal endpoint (CV death, nonfatal myocardial infarction and/or stroke, hospitalization for heart failure, and renal replacement therapy) and also for the renal endpoint alone. While in patients without CVD no relationship was found, in the subgroup with CVD history at baseline significant association between RHR and the incidence of CV and/or renal events was ascertained [9]. However, “hard” renal end-point, an end-stage renal disease (ESRD), is impractical in majority of observational cohorts and interventional studies due to relatively short follow-up. Furthermore, diabetic kidney disease (DKD) appears to be phenotypically heterogeneous (see further) and thus pathways and mediators (e.g., RHR) leading to ESRD might differ.

As documented by recent studies in both types of diabetes, progressive renal decline (defined as continuous rate of glomerular filtration rate (GFR) loss ≥ 3.3% per year) might coexist with a “classical” form of DKD with increased urinary albumin excretion preceding GFR decline [10–12]. No study, so far, focused on predictive power of RHR for DKD progression considering both phenotypes (albuminuric versus nonalbuminuric DKD) in T2DM patients.

Therefore, the aims of the present study were (1) to evaluate whether RHR is associated with DKD stage or CVD at baseline, (2) to eventually replicate in our cohort of T2DM patients previous sporadic positive findings on RHR as a predictor of CVD and DKD endpoints and death in T2DM patients, and finally (3) to specifically address RHR predictive potential for progressive renal decline in our cohort.

## 2. Materials and Methods

### 2.1. Subjects

A total of 421 T2DM patients (unrelated Caucasian subjects from South Moravia region, Czech Republic), 51.5% of men, with median age 67 [IQR 61–75], median DM duration 14 years [IQR 8–21], and range of DKD stages at baseline, were enrolled into the study between 2002 and 2010. Prospective data were collected until 2013.

Severity of DKD was defined according to the urinary albumin excretion (UAE) and stage of chronic kidney disease (CKD) by GFR assessed by creatinine clearance based on 24 h urine collection. Both parameters, UAE and GFR, were repeatedly measured at least once in 6 months or more often; staging for DKD and CKD was based on two consecutive values. At baseline, the study sample consisted of normoalbuminuric subjects (UAE < 30 mg/24 h, 8.8%), microalbuminuric subjects (UAE 30–300 mg/24 h, 30.4%), macroalbuminuric subjects (UAE > 300 mg/24 h, 51.5%), and subjects with end-stage renal disease (ESRD, 9.3%). Respective staging for CKD in the same sample was CKD I (GFR ≥ 90 mL/min per 1.73 m^2^, 17.3%), CKD II (60–89 mL/min per 1.73 m^2^, 18.3%), CKD III (30–59 mL/min per 1.73 m^2^, 36.9%), CKD IV (15–29 mL/min per 1.73 m^2^, 16.3%), and subjects with CKD V at baseline (GFR < 15 mL/min per 1.73 m^2^ or maintenance haemodialysis, 11.2%). Progressive renal decline was defined as a negative change of GFR equal to or steeper than 3.3% per year and the patient is referred to as a “decliner” and the rest as “nondecliners.” Cut-off of GFR loss ≥ 3.3% per year has been used in previous reports [10, 11] and corresponds to the 2.5th percentile of the distribution of annual renal function loss in a general population [13]. A history of DKD at baseline (DKD-b^+^) was defined as GFR < 60 mL/min/1.73 m^2^ or macroalbuminuria. A history of CVD at baseline (CVD-b^+^) was defined as a history of coronary artery disease, nonfatal myocardial infarction or stroke, lower limb amputation, or revascularization. RHR at baseline was determined either by 1 minute radial artery palpation or from ECG records. For detailed description of the whole group and CVD-b^+^ and CVD-b^−^ or DKD-b^+^ and DKD-b^−^ subgroups see Table 1.

Informed consent was obtained from each patient prior to being included in the study. The study was performed according to the recommendations of the Declaration of Helsinki and approved by the Ethical Committee of Medical Faculty, Masaryk University Brno.

### 2.2. Follow-Up

Subjects were prospectively followed for a median of 43 [22–77] months in diabetes and nephrology units of the two university hospitals in Brno and following end-points were considered as (1) progression of DKD defined as a decline of GFR < 60 mL/min per 1.73 m^2^ during the follow-up period for those with GFR ≥ 60 mL/min per 1.73 m^2^ at baseline or achieving ESRD, development of overt macroalbuminuria in normo- and microalbuminuric subjects at baseline, or progression of CKD by at least stage for those with CKD III and IV at baseline, (2) major adverse cardiovascular event (MACE), that is, fatal or nonfatal myocardial infarction or stroke, lower limb amputation, or revascularization, and (3) all-cause mortality (ACM). Only non-ESRD/non-CKD V at inception patients with complete follow-up information were included in the time-to-event analysis; that is, a total of 376 subjects were considered with the 48 month follow-up median [28–79].

### 2.3. Statistical Analysis

Data are expressed as median [interquartile range, IQR] or as percentages. Differences in continuous variables between the groups were analysed using Mann-Whitney test. Kaplan-Meier curves with log-rank testing were applied for time-to-event analysis to analyse the effect of RHR categories (< and ≥ actual median RHR and arbitrary cut-off 65 bpm) on studied outcomes. Standard competing risk methodology focusing on cumulative incidence was adopted for the nonparametric estimation and modelling of associations of the potential risk factors and the progression of DKD, MACE, and death. Gray test [14] was used to assess the differences in cumulative incidence of the competing risks with respect to the risk factors, and Fine and Gray model [15] was used to evaluate the predictive potential of the considered parameters. For all standard analysis, Statistica for Windows (Statsoft Inc., Tulsa, OK, USA) was used. *P* ≤ 0.05 was considered statistically significant.

## 3. Results

### 3.1. Analysis of Baseline Data

A history of the CVD was present in 81 (19.2%) patients at baseline; DKD (GFR < 60 mL/min or proteinuria) was present in 328 (77.9%) at baseline. For characteristics of subjects, see Tables 1 and 2.

In a CVD-b^+^ subgroup of patients (groups divided according to having or not having CVD at baseline), they were more frequently men, significantly older, and with a longer diabetes duration, higher fasting plasma glucose (FPG) levels, higher urea and creatinine plasma levels, and higher degree of albuminuria, while they did not differ in RHR, systolic (SBP) and diastolic (DBP) blood pressure, and HbA1c levels compared to CVD-b^−^ subgroup. Finally there was no significant difference in the presence of a renal history at baseline and a proportion of decliners between subgroups.

When the patients were divided according to having or not having DKD at baseline, DKD-b^+^ subjects had (similarly to CVD-b^+^) higher age, longer diabetes duration, higher urea and creatinine plasma levels and higher degree of albuminuria, additionally higher DBP and higher frequency of CVD at baseline, and finally higher proportion of decliners. There was no difference in sex, FPG, HbA1c levels, SBP, and again RHR.

Comparisons of possible differences in RHR in the presence or absence of the treatment with beta-blockers, renin-angiotensin-aldosterone system blockers (i.e., sartans or angiotensin 1 receptor, 2 blockers), or other antihypertensive therapy (i.e., diuretics, Ca-blockers, and central antihypertensive drugs) revealed no statistically significant differences in the whole group or any of the CVD-b^+/−^ or DKD-b^+/−^ subgroups (all *P* > 0.05, Mann-Whitney test). For frequencies of each drug group prescription within the whole group or subgroups, see Tables 1 and 2 (all *P* > 0.05, chi-square test).

Finally, we assessed correlations of RHR with other clinical data (age, diabetes duration, SBP, DBP, FPG, HbA1c, creatinine, urea, and total cholesterol) and found significant correlations with FPG (*r* = 0.12, *P* = 0.02, Spearman) and SBP (*r* = 0.16, *P* = 0.028, Spearman).

### 3.2. Follow-Up Analysis

*(A) Kaplan-Meier Analysis of Separate End-Points*. During the follow-up period, cumulative incidences of DKD progression, MACE, and all-cause mortality were 48.3%, 23.1%, and 38.9%, respectively, in the whole group; for incidences in CVD-b^+/−^ or DKD-b^+/−^ subgroups separately, see Table 3. Of a total of 376 subjects analysed in the follow-up study, 62 had both CVD and DKD at baseline. Of those, 35 (56%) died and 27 (44%) survived (*P* > 0.05, chi-square test). Median RHR was 75 bpm in the whole group. Furthermore, 191 (45.4%) of patients were found as GFR decliners. No statistically significant difference in RHR was ascertained between decliners and nondecliners (*P* > 0.05, Mann-Whitney test).

Irrespective of non-significant differences in RHR between subjects with CVD or DKD at baseline (b^+^) compared to those without (b^−^), analyses were still performed for (i) the whole group and (ii) CVD-b^+/−^ and (iii) DKD-b^+/−^ subgroups. Using time-to-event analyses, any significant differences in the cumulative incidence of the three studied outcomes were found between RHR </≥75 bpm (i.e., our median) and 65 bpm (i.e., arbitrary cut-off used in majority of meta-analyses [3]) neither in the whole group nor in the CVD-b^+/−^ or DKD-b^+/−^ subgroups (*P* > 0.05, log-rank test).

In spite of the fact that RHR did not significantly differ between beta-blocker users and nonusers in the whole group or any of the subgroups defined based on CVD or DKD status at baseline, we still analysed the effect of RHR (cut-off </≥65 or 75 bpm) in beta-blocker naive subjects separately (*n* = 145). No significant differences were assessed using this subpopulation analysis (all *P* > 0.05, log-rank test).

*(B) Competing Risk Analysis*. Since estimates based on the naive Kaplan-Meier curves do not consider the presence of competing risks, they apparently tend to overestimate the probability of occurrence of the individual events in time. We compared groups with initial RHR </≥ median (75 bpm) or </≥ arbitrary cut-off (65 bpm), respectively. *P* values in univariate analysis of competing risk model did not indicate a significant effect of the RHR on the cumulative incidence of the two competing risks (i.e., DKD progression and MACE, *P* > 0.05, Gray test). Similarly, no significance was found for all-cause death (*P* > 0.05, Gray test).  Table 4 shows the results of a Fine and Gray model. Again, no significant difference between groups was found.

## 4. Discussion and Conclusions

In the present study we evaluated a predictive potential of baseline RHR for progression of DKD (and more specifically for rapid GFR decline), MACE, and all-cause death in T2DM patients. In the cross-sectional part of our study, we compared clinical and biochemical data between groups of diabetic subjects with or without initial DKD or CVD. Subjects in both DKD-b^+^ and CVD-b^+^ subgroups had significantly higher age, longer diabetes duration, worse renal parameters (higher levels of urea and creatinine and higher degree of albuminuria), higher FPG, and male predominance in a CVD-b^+^ subgroup, while DKD-b^+^ subgroup had higher DBP and a higher proportion of decliners.

Although previous studies found an association between RHR and prevalence of baseline CVD [7–9] or DKD [8, 9] in T2DM patients, we were not able to ascertain similar significant differences in RHR in any of those categories studied. The major focus of the study was the prospective evaluation of the predictive potential of RHR for the MACE and progression of DKD. Moreover, we believe, this is a first study dealing with RHR in relation to the progressive renal decline. The concept of progressive renal decline was proposed by Krolewski in T1DM patients as an alternative pathway to albuminuric DKD [16]. Pugliese et al. in the RIACE study on T2DM patients [12] found a reduced estimated GFR (eGFR) without albuminuria independently associated with a significant CVD burden, higher than albuminuria alone, whereas the combination of reduced eGFR and albuminuria marked a further increased risk of CVD events in an additive manner. We found a higher proportion of decliners in DKD-b^+^ subgroup of patients, which could be explained by generally nonlinear pattern of renal disease progression; however, no such difference was found between CVD-b^+^ and CVD-b^−^ subgroups in spite of the fact that CVD-b^+^ group had worse renal parameters at baseline similarly to DKD-b^+^. This might signify a specific pathogenic mechanism unrelated to CVD and this topic warrants further study.

Since beta-blockers or RAAS blockers have an obvious influence on RHR, we adjusted our analyses for the therapy modality. There was no therapy-related effect on any of the outcomes studied and on any of subgroups.

Our finding of positive correlation of RHR with FPG and SBP corresponds with results of previous studies; for example, in a large study of a French population with almost 100,000 participants, heart rate was positively associated with blood pressure, triglycerides, glycaemia, and physical inactivity and negatively with body height [17].

In the prospective part of the study, we were unable to identify any significant relationship of an initial RHR with DKD progression, major adverse cardiovascular event, and all-cause mortality in our cohort. Since more than one end-point may occur in the same patient, a competing risk methodology for multiple risk scenario was used. Yet again, RHR was not identified as a significant risk factor for DKD progression or MACE in the univariate competing risk model. Those findings are contrary to results of previous sporadic studies. A prospective study by Hillis et al. found in a cohort of 11,140 T2DM patients with a history of CVD participating in ADVANCE study [18] an association between higher baseline RHR and a greater risk of developing microvascular endpoint (defined as a composite of new or worsening nephropathy) during 4.4-year follow-up. After adjustment for age, sex, and randomized treatment (perindopril-indapamide), a 10 bpm increase in baseline RHR was associated with an 18% increase in the observed hazard [8]. Another recent study of 1088 T2DM patients by Miot et al. [9] focused on both CV and renal parameters (briefly, 31% of patients had a history of CVD at baseline (CVD-b^+^) and median of follow-up was 4.2 years; mean RHR was 67.7 bpm in CVD-b^+^ subgroup and 72.4 bpm in CVD-b^−^ subgroup) but not considering the drug therapy in the analyses ascertained RHR associated with the incidence of CV and renal morbidity/mortality (*P* = 0.0002) and also with renal risk alone adjusted for all-cause death as a competing event in the CVD-b^+^ subgroup only (*P* < 0.0001). In the CVD-b^−^ subgroup, no relation was found between RHR and the incidence of CV and/or renal events. We have not been able to replicate any predictive effect of the RHR for the renal or CV outcomes in T2DM population of Czech Republic. Given similar settings of our study, one of the possible explanations might certainly be a smaller sample size, slightly shorter follow-up, different definition of endpoints, or different cut-offs for RHR. Regarding the latter, of plethora of possibilities, we have chosen stratification according to two RHR cut-offs, a median RHR and an arbitrary cut-off 65 bpm in line with results of a meta-analysis by Woodward et al. [3].

There are several pathogenic mechanisms proposed by which an elevated heart rate might mediate development and progression of DKD and CVD. It has been suggested that a higher heart rate might promote microalbuminuria because of increased exposure of the glomerulus to arterial pressure waves [19]. An increased heart rate has also variety of direct detrimental cardiovascular consequences including endothelial dysfunction and atherogenic activity that are important factors in the progression of DKD too [20]. A higher heart rate also is associated with factors such as obesity, higher blood pressure, atherogenic dyslipidaemia, and reduced physical activity [21], all of which are associated with an increased risk of microvascular complications and are targets for intervention to improve outcome in patients with diabetes mellitus [22]. Finally, a faster resting heart rate is a characteristic feature of autonomic neuropathy, which is in turn associated with an increased prevalence of other complications, such as DKD or retinopathy [23]. Therefore, it is conceivable that mechanisms listed could have synergistic effects and represent potentially very important pathogenic mechanism; on the contrary, the effect might operate in stage-dependent fashion given our negative finding of increased RHR as a general predictor of DKD progression in T2DM.

We are of course aware of several limitations of our study potentially impacting on its negative outcome. First of all, current sample size is relatively small compared to previous studies. This together with the rather high representation of subjects with baseline DKD or CVD might weaken the potential predictive power of RHR in the situation of more advanced stages of cardiovascularly relevant comorbidities. Therefore, although our results indicate several trends—for example, patients with a history of CVD or DKD at baseline had more frequently beta-blockers in therapy and CVD-b^+^ patients have a tendency to a higher RHR—the results were not found statistically significant in our cohort.

In conclusion, recent study analysing the potential of RHR for the prediction of progression of DKD (and specifically progressive renal decline), major cardiovascular event, and all-cause death in a cohort of Caucasian T2DM subjects did not reveal significant effect (not even in the subgroup of heart rate affecting therapy-naïve subjects). Additional studies are therefore warranted to decipher event. Additional studies are therefore warranted to decipher if RHR could be an applicable risk marker for DKD.

## Figures and Tables

**Table 1:** Clinical and biochemical characteristics of subjects divided according to history of CVD, baseline data.

Variables	All	CVD-b^+^	CVD-b^−^	*P*
*n* (%)	421	81 (19.2)	340 (80.0)	
Sex: men/women, *n* (%)	217 (52)/204 (48)	52 (64)/29 (36)	165 (49)/175 (51)	0.01
Age (years)	67 [61–75]	70 [63–76]	67 [60–74]	0.009
Diabetes duration (years)	14 [8–21]	16 [12–23]	13 [7–20]	1 × 10^−4^
FPG (mmol/L)	8.5 [6.8–10.9]	9.3 [7.8–11.8]	8.1 [6.6–10.8]	0.02
HbA1c (%), IFCC calibration	6.4 [5.4–8.1]	6.6 [5.9–8.0]	6.4 [5.2–8.1]	NS
Creatinine (*μ*mol/L)	142 [114–214]	188 [139–311]	135 [101–191]	<1 × 10^−6^
Urea (mmol/L)	11.0 [7.3–17.3]	16.0 [10.1–21.6]	9.9 [6.9–16.2]	<1 × 10^−5^
Albuminuria (mg/24 hours)	500 [140–2080]	1540 [350–3670]	400 [130–1740]	7 × 10^−4^
Systolic blood pressure (mmHg)	144 [130–160]	145 [125–150]	142 [130–160]	NS
Diastolic blood pressure (mmHg)	80 [75–90]	80 [70–95]	80 [75–90]	NS
Total cholesterol (mmol/L)	4.9 [4.2–5.8]	4.5 [3.9–5.3]	4.9 [4.3–5.8]	0.02
Beta blockers users (%)	46.3	50.6	45.3	NS
RAAS inhibitors users (%)	62	53.1	64.1	NS
Other antihypertensive therapy (%)	69.1	71.6	68.5	NS
History of renal disease, *n* (%)	**328 (77.9)**	**73 (90)**	**255 (75)**	**NS**
Decliners (GFR loss ≥ 3.3% per year), *n* (%)	**191 (45.4)**	**36 (44)**	**155 (45.6)**	**NS**
RHR (bpm)	75 [70–80]	75 [70–84]	75 [70–80]	NS

Data are expressed as median [interquartile range] or percentages. Differences evaluated by nonparametric Mann-Whitney or chi-square test, respectively.

FPG, fasting plasma glucose; HbA1c, glycated haemoglobin; GFR, glomerular filtration rate; RAAS, renin-angiotensin-aldosterone system; RHR, resting heart rate.

**Table 2:** Clinical and biochemical characteristics of subjects divided according to history of DKD, baseline data.

Variables	All	DKD-b^+^	DKD-b^−^	*P*
*n* (%)	421	328 (77.9)	93 (22.1)	
Sex: men/women, *n* (%)	217 (52)/204 (48)	175(53)/153 (47)	42 (45)/51 (55)	NS
Age (years)	67 [61–75]	68 [62–75]	63 [56–71]	3 × 10^−4^
Diabetes duration (years)	14 [8–21]	15 [9–22]	10 [6–15]	4 × 10^−5^
FPG (mmol/L)	8.5 [6.8–10.9]	8.8 [6.9–11.2]	8.0 [6.8–9.7]	NS
HbA1c (%), IFCC calibration	6.4 [5.4–8.1]	6.5 [5.5–8.1]	6.2 [5.3–8.0]	NS
Creatinine (*μ*mol/L)	142 [114–214]	164 [125–258]	91 [81–107]	<1 × 10^−6^
Urea (mmol/L)	11.0 [7.3–17.3]	13.3 [8.7–19.6]	6.1 [5.3–7.6]	<1 × 10^−6^
Albuminuria (mg/24 hours)	500 [140–2080]	840 [260–2350]	110 [90–150]	<1 × 10^−6^
Systolic blood pressure (mmHg)	144 [130–160]	144 [130–160]	140 [130–160]	NS
Diastolic blood pressure (mmHg)	80 [75–90]	80 [74–90]	90 [80–98]	0.001
Total cholesterol (mmol/L)	4.9 [4.2–5.8]	4.9 [4.2–5.7]	4.9 [4.3–5.9]	NS
Beta blockers at treatment (%)	46.3	48.5	33.3	NS
RAAS inhibitors at treatment (%)	62	60.7	58.1	NS
Other antihypertensive therapy (%)	69.1	71	52.7	NS
History of CV disease, *n* (%)	**328 (77.9)**	**73 (22)**	**8 (9)**	**0.01**
Decliners (GFR loss ≥ 3.3% per year), *n* (%)	**191 (45.4)**	**164 (50)**	**27 (29)**	3 × 10^−4^
RHR (bpm)	75 [70–80]	74 [70–80]	74 [70–80]	NS

Data are expressed as median [interquartile range] or percentages. Differences evaluated by nonparametric Mann-Whitney or chi-square test, respectively.

CV, cardiovascular; FPG, fasting plasma glucose; HbA1c, glycated haemoglobin; GFR, glomerular filtration rate; RAAS, renin-angiotensin-aldosterone system; RHR, resting heart rate.

**Table 3:** Cumulative incidence of DKD progression, MACE and all-cause mortality in the whole group and subgroups.

	DKD progressor	*P*	MACE	*P*	ALL-cause mortality	*P*
Whole group (*n* = 376)	48.3%	—	23.1%	—	38.9%	—

CKD-b^+^ (*n* = 66)	51.5%	NS	22.3%	NS	53.0%	NS
CKD-b^−^ (*n* = 310)	47.1%		27.3%		36.1%	

DKD-b^+^ (*n* = 283)	53.4%	0.047	26.8%	0.023	48.4%	<1 × 10^−4^
DKD-b^−^ (*n* = 93)	26.9%		11.7%		6.5%	

**(a):** 

Risk factor	Risk category	Basal category	Fine and Gray model		
			HR	95% CI	*P* value
RHR	≥65 bpm	<65 bpm	1.11	0.77–1.60	0.580
RHR	≥75 bpm	<75 bpm	0.97	0.73–1.29	0.820

**(b):** 

Risk factor	Risk category	Basal category	Fine and Gray model		
			HR	95% CI	*P* value
RHR	≥65 bpm	<65 bpm	0.79	0.51–1.23	0.300
RHR	≥75 bpm	<75 bpm	0.87	0.60–1.27	0.470

## References

[1] Palatini P., Julius S. (2004). Elevated heart rate: a major risk factor for cardiovascular disease. *Clinical and Experimental Hypertension*.

[2] Fox K., Borer J. S., Camm A. J. (2007). Resting heart rate in cardiovascular disease. *Journal of the American College of Cardiology*.

[3] Woodward M., Webster R., Murakami Y. (2014). The association between resting heart rate, cardiovascular disease and mortality: evidence from 112,680 men and women in 12 cohorts. *European Journal of Preventive Cardiology*.

[4] Lang C. C., Gupta S., Kalra P. (2010). Elevated heart rate and cardiovascular outcomes in patients with coronary artery disease: clinical evidence and pathophysiological mechanisms. *Atherosclerosis*.

[5] Stettler C., Bearth A., Allemann S. (2007). QTc interval and resting heart rate as long-term predictors of mortality in type 1 and type 2 diabetes mellitus: a 23-year follow-up. *Diabetologia*.

[6] Linnemann B., Janka H. U. (2003). Prolonged QTc interval and elevated heart rate identify the type 2 diabetic patient at high risk for cardiovascular death. The Bremen diabetes study. *Experimental and Clinical Endocrinology and Diabetes*.

[7] Hillis G. S., Woodward M., Rodgers A. (2012). Resting heart rate and the risk of death and cardiovascular complications in patients with type 2 diabetes mellitus. *Diabetologia*.

[8] Hillis G. S., Hata J., Woodward M. (2012). Resting heart rate and the risk of microvascular complications in patients with type 2 diabetes mellitus. *Journal of the American Heart Association*.

[9] Miot A., Ragot S., Hammi W. (2012). Prognostic value of resting heart rate on cardiovascular and renal outcomes in type 2 diabetic patients: a competing risk analysis in a prospective cohort. *Diabetes Care*.

[10] Krolewski A. S., Niewczas M. A., Skupien J. (2014). Early progressive renal decline precedes the onset of microalbuminuria and its progression to macroalbuminuria. *Diabetes Care*.

[11] Krolewski A. S., Gohda T., Niewczas M. A. (2014). Progressive renal decline as the major feature of diabetic nephropathy in type 1 diabetes. *Clinical and Experimental Nephrology*.

[12] Pugliese G., Solini A., Bonora E. (2014). Chronic kidney disease in type 2 diabetes: lessons from the Renal Insufficiency And Cardiovascular Events (RIACE) Italian Multicentre Study. *Nutrition, Metabolism and Cardiovascular Diseases*.

[13] Lindeman R. D., Tobin J., Shock N. W. (1985). Longitudinal studies on the rate of decline in renal function with age. *Journal of the American Geriatrics Society*.

[14] Gray R. J. (1988). A class of K-sample tests for comparing the cumulative incidence of a competing risk. *The Annals of Statistics*.

[15] Fine J. P., Gray R. J. (1999). A proportional hazards model for the subdistribution of a competing risk. *Journal of the American Statistical Association*.

[16] Krolewski A. S. (2015). Progressive renal decline: the new paradigm of diabetic nephropathy in type 1 diabetes. *Diabetes Care*.

[17] Morcet J.-F., Safar M., Thomas F., Guize L., Benetos A. (1999). Associations between heart rate and other risk factors in a large French population. *Journal of Hypertension*.

[18] ADVANCE Management Committee (2001). Study rationale and design of ADVANCE: action in diabetes and vascular disease—preterax and diamicron MR controlled evaluation. *Diabetologia*.

[19] Böhm M., Reil J. C., Danchin N., Thoenes M., Bramlage P., Volpe M. (2008). Association of heart rate with microalbuminuria in cardiovascular risk patients: data from I-SEARCH. *Journal of Hypertension*.

[20] Nakagawa T., Tanabe K., Croker B. P. (2011). Endothelial dysfunction as a potential contributor in diabetic nephropathy. *Nature Reviews Nephrology*.

[21] Palatini P. (2009). Elevated heart rate in cardiovascular diseases: a target for treatment?. *Progress in Cardiovascular Diseases*.

[22] American Diabetes Association (2008). Standards of medical care in diabetes—2008. *Diabetes Care*.

[23] Vinik A. I., Maser R. E., Mitchell B. D., Freeman R. (2003). Diabetic autonomic neuropathy. *Diabetes Care*.

